# *Akkermansia muciniphila* - friend or foe in colorectal cancer?

**DOI:** 10.3389/fimmu.2023.1303795

**Published:** 2023-12-06

**Authors:** Ekaterina O. Gubernatorova, Ekaterina A. Gorshkova, Marina A. Bondareva, Olga A. Podosokorskaya, Anna D. Sheynova, Anastasia S. Yakovleva, Elizaveta A. Bonch-Osmolovskaya, Sergei A. Nedospasov, Andrey A. Kruglov, Marina S. Drutskaya

**Affiliations:** ^1^ Engelhardt Institute of Molecular Biology, Russian Academy of Sciences, Moscow, Russia; ^2^ Center for Precision Genome Editing and Genetic Technologies for Biomedicine, Engelhardt Institute of Molecular Biology, Russian Academy of Sciences, Moscow, Russia; ^3^ Belozersky Institute of Physico-Chemical Biology Lomonosov Moscow State University, Moscow, Russia; ^4^ German Rheumatism Research Center (DRFZ), Leibniz Institute, Berlin, Germany; ^5^ Winogradsky Institute of Microbiology, Research Centre of Biotechnology Russian Academy of Sciences (RAS), Moscow, Russia; ^6^ Faculty of Biology, Lomonosov Moscow State University, Moscow, Russia; ^7^ Division of Immunobiology and Biomedicine, Sirius University of Science and Technology, Federal Territory Sirius, Krasnodarsky Krai, Russia

**Keywords:** intestinal inflammation, colorectal cancer, mucin-reducing bacteria, Akkermanisa municiphila, probiotic

## Abstract

*Akkermansia muciniphila* is a gram-negative anaerobic bacterium, which represents a part of the commensal human microbiota. Decline in the abundance of *A. muciniphila* among other microbial species in the gut correlates with severe systemic diseases such as diabetes, obesity, intestinal inflammation and colorectal cancer. Due to its mucin-reducing and immunomodulatory properties, the use of probiotics containing *Akkermansia* sp. appears as a promising approach to the treatment of metabolic and inflammatory diseases. In particular, a number of studies have focused on the role of *A. muciniphila* in colorectal cancer. Of note, the results of these studies in mice are contradictory: some reported a protective role of *A. muciniphila* in colorectal cancer, while others demonstrated that administration of *A. muciniphila* could aggravate the course of the disease resulting in increased tumor burden. More recent studies suggested the immunomodulatory effect of certain unique surface antigens of *A. muciniphila* on the intestinal immune system. In this Perspective, we attempt to explain how *A. muciniphila* contributes to protection against colorectal cancer in some models, while being pathogenic in others. We argue that differences in the experimental protocols of administration of *A. muciniphila*, as well as viability of bacteria, may significantly affect the results. In addition, we hypothesize that antigens presented by pasteurized bacteria or live *A. muciniphila* may exert distinct effects on the barrier functions of the gut. Finally, *A. muciniphila* may reduce the mucin barrier and exerts combined effects with other bacterial species in either promoting or inhibiting cancer development.

## Introduction

Gut microbiota plays an important role in maintaining intestinal homeostasis. Among a huge variety of gut colonizing bacteria, *Akkermansia muciniphila* (*A. muciniphila)* deserves special attention. *A. muciniphila* is a non-motile gram-negative mucin-degrading bacterium of the phylum *Verrucomicrobiota* first isolated from human faeces by Derrien et al. (1, 2). A strict anaerobe, *A. muciniphila* adapted to living in human intestine by producing mucin-degrading enzymes (α- and β-D-galactosidase, α-L fucosidase and other) to utilize mucins as a source of nitrogen and carbon (3, 4). In mucin-depleted culturing conditions *A. muciniphila* is capable of switching to glucose-driven glycolysis (5), thus utilizing the excess of glucose. Also, it was demonstrated that *A. muciniphila* utilizes circulating host lactate and urea (6), reshaping host systemic metabolism.

*A. muciniphila* is localized mostly in the colon mucus layer of healthy individuals with relative abundance of 3% (7). Akkermansia-like sequences were found in other anatomical regions of the human digestive tract and even in breast milk (7). *A. muciniphila* was reported as a part of commensal microbiota in other animal species, including mice (8–10), making mice a convenient animal model to study the *in vivo* functions of this microorganism (11, 12).

Over the past ten years, numerous studies addressed the role of *A. muciniphila* in health and disease. Reduced amounts of *A. muciniphila* were reported in obesity and type 2 diabetes (13) and are associated with Western-type diet. The same correlation was shown for a high-fat (14, 15) and high-sucrose diet in mice (16). It was also established that the decline in *A. muciniphila* abundance correlated with the development of intestinal inflammation, colorectal cancer, and even with cognitive disorders such as depression and anxiety (17). Thus, a promising therapeutic potential of *A. muciniphila* as a probiotic or postbiotic and gut microbiota modulator is widely recognized (18–20). However, several studies suggest that *A. muciniphila* over representation may correlate with negative prognosis of anti-cancer therapy (21). Animal studies aimed to elucidate the specific molecular mechanisms of *A. muciniphila* effects in colorectal cancer remain contradictory. In this regard, we hypothesized that the introduction of high doses of *Akkermansia* can lead to disruption of homeostasis and increased tumor growth, while moderate and gentle introduction of bacteria has a protective effect. In present article we are attempting to explain how the differences in experimental settings may affect the results in these earlier reported studies.

## Regulatory of effects of *A. muciniphila* on gut homeostasis

Microbiota interacts with the immune system either directly by activating the immune cells or via production of immunomodulatory metabolites and other molecules. Recent studies suggested that *A. muciniphila* acquired mechanisms to control host metabolism in the gut and, therefore, may contribute to healthy niche maintenance. For example, protein P9 secreted by *A. muciniphila* was reported to directly promote the production of GLP-1 by the human primary intestinal epithelial cells, stimulating insulin production and fat browning (22). The most abundant outer membrane pili protein of *A. muciniphila*, Amuc_1100, was shown to provide beneficial effects in HFD-mice (23). As TLR2 activator, Amuc_1100 demonstrated effects on immune cells (24–26). Another study reported that Amuc_1100 synthesis was increased in mucin-depleted conditions (5), while Khan et al. found that increased sugar consumption in mice may lead to overgrowth of *A. muciniphila* within 1 week and its mucin-degrading activity may result in thinning of the mucus layer (27).

Bae et al. identified a lipid from *A. muciniphila’s* cell membrane, diacyl phosphatidylethanolamine with two branched chains (a15:0-i15:0 PE), that can contribute to immunomodulatory activity of bacteria in TLR2-TLR1 dependent manner. Interestingly, in high doses it triggers the release of TNF and IL-6 but not IL-10 or IL-12p70 by mouse BMDCs, while in low doses it resets activation thresholds and responses for immune signaling, so that weak activating signals are ignored and strong signals are moderated, contributing to the regulation of immune response (28).

A newly described outer membrane protein, Amuc_2172, was implicated in activation of immune cells via promotion of HSP70 production in cellular microenvironments (29). Bacterial control of the host immune system may indirectly affect barrier integrity, as well as suppression of autoimmunity against symbionts. It was shown that *A. muciniphila* secretes tripeptide RKH (Arg-Lys-His), which binds to TLR4 block signal transduction, rescuing mice form lethality in a model of CLP-induced sepsis (30). RKH production represents direct immune-suppressing activity of *A. muciniphila*. Since inflammation plays a significant role in cancer development, a proper use of the evolutionarily selected functions of *A. muciniphila* in its interaction with the host may represent novel therapeutic strategies to control inflammation and tumorigenesis.

## *A. muciniphila* in gut inflammation control

Both colorectal cancer and inflammation are influenced by many factors, such as heredity, habits and nutrition, but in recent years much attention was paid to the relationship between the microbiota and the host immune system. The development of inflammatory bowel disease correlates with an increase in opportunistic microorganisms and a decrease in beneficial *Bifidobacteria* and *Lactobacilli* (31, 32). Colonization by *A. muciniphila* is thought to occur early in life during the induction of RORγt^+^Foxp3^+^ Tregs to ensure intestinal homeostasis (33). At the same time, a decrease in the abundance *of A. muciniphila* is characteristic for inflammatory bowel diseases (34–36), as well as for dysbiosis associated with cancer (25).

Studies of *A. muciniphila* in mouse models of intestinal inflammation suggested a protective role of this bacterium or its derivatives (**Table 1**). For example, the administration of viable *A. muciniphila* in both low (10^8^ CFU) and high (3×10^9^ CFU) doses reduced the severity of colitis, increased mucus production (37, 38, 40), reduced the intensity of inflammation (40, 41), and also compensated for dysbiosis associated with inflammation (39). Furthermore, administration of pasteurized bacterium, which exemplifies the concept of “postbiotic” or a preparation of inanimate microorganisms and their components that confers a health benefit on the host (49), as well as recombinant Amuc_1100 or Amuc_2109, also reduced cytotoxic cell accumulation in the intestine and NRLR3 activation, suggesting strong antigenic properties of this bacterium (25, 42). Other data, on the contrary, indicate that the introduction of live bacteria aggravates the symptoms of colitis, however, the same studies showed the protective role of extracellular vesicles of *A. muciniphila* (29, 44). Since inflammation is the key factor in the development of colorectal cancer, and *A. muciniphila* has been shown to be an effective anti-inflammatory agent, the bacterium is considered a promising probiotic that can reduce the development of cancer.

**Table 1:** Effects of *A. muciniphila* in mouse models of acute and chronic intestinal inflammation and gastrointestinal cancer.

Effects of *A. muciniphila* in mouse models of acute and chronic intestinal inflammation						
#	Bacteria introduction protocol	Form of bacteria or antigen	Dose of bacteria	Effect	Colitis induction	Reference
1	Oral administration daily for 5 days during colitis induction	**Viable** *A. muciniphila* or outer membrane vesicles from *A. muciniphila*	10^8^ CFU in 100 mcl per mouse or 20 mcg *A. muciniphila* OMVs in 100 mcl per mouse	Reduced colonic inflammation with **increased production of mucus**	7 days of 5% DSS	(37)
2	Oral administration daily for 14 days before colitis induction and after **antibiotic treatment**	**Viable** *A. muciniphila*	10^8^ CFU in 100 mcl per mouse	Alleviated colitis severity and depression-like symptoms with more **intensive mucus production and *Muc2* expression**	7 days of 2,5% DSS after psychological stress (restraining)	(38)
3	Oral administration daily 7 days before colitis induction and during colitis induction	**Viable** *A. muciniphila*	3×10^9^ CFU in 200 mcl per mouse	Ameliorated disease severity with enhanced barrier function and **alleviated colitis-induced dysbiosis**	7 days of 2% DSS	(39)
4	Oral administration daily for 7 days after **antibiotic treatment** and before colitis induction	**Viable** *A. muciniphila*	10^9^ CFU in 300 mcl per mouse	Ameliorated disease severity and body weight loss with inhibited expression of inflammatory cytokines and **higher NRLP3 activation**	8 days of 3% DSS	(40)
5	Oral administration daily during **chronic colitis induction**	**Viable** *A. muciniphila* ATCC BAA-835 strain and isolated 139 substrain	2×10^8^ CFU in 200 mcl per mouse	Improved clinical parameters including spleen weight, colon inflammation index, and colon histological score with decreased expression of inflammatory cytokines and fecal lipocalin-2. ATCC BAA-835 strain was more powerful in amelioration of inflammation than murine substrain 139	Three cycles of 3 days of 3% DSS	(41)
6	Oral administration daily 14 days before the colitis induction till sacrifice	**Pasteurized** *A. muciniphila* or **recombinant surface protein Amuc_1100**	1.5×10^8^ CFU in 100 mcl per mouse Or 3 mcg of protein in 100 mcl per mouse	Reduced colonic inflammation with **decreased** proportion of **CTLs** in colon	8 days of 2% DSS	(25)
7	Oral administration daily 21 days before colitis induction and during colitis induction	**Recombinant protein Amuc_2109** from *A. muciniphila*	100 mcg/kg per mouse	Ameliorated disease severity and body weight loss with **inhibited expression of inflammatory cytokines and NRLP3 activation**	7 days of 2% DSS	(42)
8	Oral administration daily for 14 days after **antibiotics treatment**	**Viable** *A. muciniphila*	10^9^ CFU per mouse	**Increased the levels of M1-like monocytes** (CD45^+^Ly6C^+^MHCII^+^) in colon, blood, and bone marrow	7 days of 3% DSS after antibiotics	(43)
9	Oral administration daily after colitis induction till sacrifice	**Viable** *A. muciniphila* or **secreting extracellular vesicles** from *A. muciniphila*	5×10^8^ CFU in 1 ml per mouse or 100 mg in 1 ml per mouse of secreting extracellular vesicles	More severe body weight loss with *A. muciniphila* introduction and attenuated weight loss with secreting extracellular vesicles introduction	10 days of 3% DSS	(29)
10	Oral administration daily during colitis induction till sacrifice	**Viable** *A. muciniphila* or **extracellular vesicles** from *A. muciniphila*	5×10^8^ CFU or 100 mg of extracellular vesicles per mouse	More severe body weight loss with *A. muciniphila* introduction and attenuated weight loss with extracellular vesicles introduction	5 days of 2% DSS	(44)
Differential effects of *A. muciniphila* in mouse models of gastrointestinal cancer						
#	Model of cancer	Bacteria introduction protocol	Form of bacteria or antigen	Dose of bacteria	Effect	Reference
1	AOM/DSS-induced colitis-associated colorectal cancer	Oral administration at the 0, 3, 5, and 7 days of experiment before cancer induction	**Viable** *A. muciniphila*	**High dose** (10^9^ CFU) in 100 mcl per mouse	Increased number of colon tumors, more colon damage, **increased expression of inflammation markers, decreased mucus production**	(45)
2	AOM/DSS colitis-associated colorectal cancer	Oral administration every day after **antibiotic treatment** from 3 days before the DSS treatment to sacrifice but skipped the DSS treatment period	**Viable** *A. muciniphila*	**High dose** (3×10^9^ CFU) in 200 mcl per mouse	Increased number of colon tumors, impaired gut barrier function, **increased expression of inflammation markers, decreased mucus production**	(46)
3	Spontaneous tumorigenesis in *Apc^15lox^* ^/+^ mice	Oral administration three times started at 4 weeks of age after 1 week of **antibiotic treatment** till sacrifice	**Viable** *A. muciniphila*	**High dose** (10^9^ CFU) in 100 mcl per mouse	Increased number of tumors, but **more intensive mucus production**	(47)
4	AOM/DSS colitis-associated colorectal cancer	Oral administration daily 14 days before the cancer induction till sacrifice	**Pasteurized** *A. muciniphila* or **recombinant surface protein Amuc_1100**	**Low dose** (1.5×10^8^ CFU) in 100 mcl per mouse or 3 mcg of protein in 100 mcl per mouse	Decreased number of colon tumors with **expanded CTLs** in the colon and MLN	(25)
5	AOM/DSS colitis-associated colorectal cancer	Oral administration daily after cancer induction till sacrifice	**Secreting extracellular vesicles** from *A. muciniphila*	100 mg in 1 ml per mouse	Decreased number of colon tumors with **increased CTLs activity**	(29)
6	Spontaneous tumorigenesis in *Apc^Min/+^ mice*	Intraperitoneal injection twice a week for 14 weeks	**Recombinant surface protein Amuc_2172**	150 mcg/kg per mouse	Decreased number of tumors with **increased CTLs**	(29)
7	Spontaneous tumorigenesis in *Apc^Min/+^* mice with two cycles of 10-day 1% DSS	Oral administration every two days for three months after **antibiotic treatment** starting from 6-8 weeks of age till sacrifice	**Viable** *A. muciniphila*	**High dose** (10^9^ CFU) in 300 mcl per mouse	Suppressed colonic tumorigenesis, decreased systemic inflammation through facilitated **enrichment of M1-like macrophages** in an NLRP3-dependent, TLR2-dependent manner	(43)
8	Subcutaneous injection of CT26 cells in BALB/c mice	Oral administration started when the tumor reaches size 100 mm^3^ performed every day until the end of the experiment along with intraperitoneal injection of anti–PD-1	**Viable** *A. muciniphila* or **outer membrane vesicles** from *A. muciniphila*	**Low dose** (10^8^ CFU) in 100 mcl per mouse or 20 mcg *A. muciniphila* OMVs in 100 mcl per mouse	Decreased tumor size with **enhanced aPD-1 therapy efficacy**	(37)
9	Subcutaneous injection of HCT116 or CT26 cells in BALB/c nude mice	Subcutaneous injection of 3×10^6^ HCT116 or CT26 cells mixed with *A. muciniphila* (MOI = 10:1)	**Viable** *A. muciniphila*	**Low dose** (3×10^7^ CFU) per mouse	Suppressed growth of implanted HCT116 or CT26 tumors	(43)
10	Subcutaneous injection of CT26 cells in BALB/c mice	Intratumor injection twice a week after cancer induction till sacrifice	**Recombinant surface protein Amuc_2172**	150 mcg/kg per mouse	Inhibited allografted tumors growth by promoting **CTLs**	(29)
11	Subcutaneous injection of CT-26 cells with FOLFOX (oxaliplatin, fluorouracil and calcium folinate) treatment	Oral administration started when the tumor reaches size 100 mm^3^ and performed every other day after **antibiotic treatment** until sacrifice	**Viable** *A. muciniphila*	**Low dose** (10^8^ CFU) per mouse	**Enhanced anti-cancer effect of FOLFOX**, presumably due to *A. muciniphila* **effect on gut metabolomics**	(48)

## *A. muciniphila* in mouse models of colorectal cancer

Studies on the role of *A. muciniphila* in mouse models of gut cancer provided contradictory results (**Table 1**). Several reports indicated that administration of *A. muciniphila* may aggravate the development of intestinal cancer. For example, Wang F*. et al.* found that administration of *A. muciniphila* prior to induction of colorectal cancer increased the number of intestinal tumors in the AOM/DSS model in correlation with a decrease in mucus production (45). In a similar study by Wang K. et al, oral gavage with *A. muciniphila* after a course of antibiotics led to increased tumor formation with a decrease in mucin expression (46). Finally, in a model of spontaneous tumor formation in Apc^15lox/+^ mice, oral gavage with *A. muciniphila* after a course of antibiotics also increased the number of tumors, but, in contrast to the data of Wang F. and Wang K., it increased mucus production (47).

At the same time, other numerous studies confirm the protective effect of the enrichment with these bacteria on colorectal cancer. In particular, oral gavage with pasteurized *A. muciniphila*, surface antigen Amuc_1100 (25) or *A. muciniphila* secretory extracellular vesicles (29) protected mice in the AOM/DSS model by increasing cytotoxic lymphocyte activity. An increase in the activity of cytotoxic lymphocytes was also shown for another *A. muciniphila* protein - Amuc_2172 in Apc^Min/+^ mice (29). Interestingly, in the Apc^Min/+^ model therapeutic administration of live *A. muciniphila* following antibiotics in the context of DSS-induced inflammation also reduced tumor burden, apparently due to a TLR2-mediated, NRLP3-dependent increase in the activity of antitumor M1 macrophages (43). The protective role of *A. muciniphila* was also shown in a number of studies using a transplantable tumor model. Therapeutic administration of bacteria or bacterial secretory vesicles *per os* reduced the growth of grafted CT26 and also enhanced the effect of anti-PD-1 therapy (37), while intratumoral administration of *A. muciniphila* (43) or Amuc_2172 (29) reduced the growth of allografts due to the activation of CD8^+^IFNy^+^ cytotoxic cells. Finally, it was established that administration of *A. muciniphila per os* enhanced the effect of the antitumor drug FOLFOX (oxaliplatin, fluorouracil and calcium folinate), and, conversely, the use of FOLFOX led to a significant increase in the *A. muciniphila* abundance in the gut (48).

Taken together, there is a major controversy over the effects of *A. muciniphila* on the development of intestinal cancer.

## The molecular form of *A. muciniphila* shapes the outcome

One of the factors potentially explaining the different effects of *A. muciniphila* in colorectal cancer may be related to different protocols of bacterial administration - from live or pasteurized *A. muciniphila* to recombinant peptides and extracellular vesicles derived from these bacteria. Thus, in all studies in which *A. muciniphila* aggravated tumor growth, live bacteria was used at high concentration of 10^9^ CFU (45–47), and in some studies, this bacteria was introduced following a course of antibiotics (46, 47). It was shown that the high dose of *A. muciniphila* after a course of antibiotics in colorectal cancer model dramatically changed the composition of the microbiota. There was no expansion of *A. muciniphila* itself, but rather an increase in the opportunistic bacteria, including *Clostridia*. This resulted in aggravation of dysbiosis and disturbance in the metabolic profile as indicated by a decrease of bile acids and short-chain fatty acids (46). Presumably, a high dose of *A. muciniphila*, especially after the depletion of gut microbiota with antibiotics, can be interpreted by the immune system as an infection and leads to an increased inflammation due to disrupted microbiota composition and increased opportunistic pathogens in the gut. Moreover, it was shown that in antibiotic treated mice, some phylogroups of *A. muciniphila* may outcompete others, affecting the outcome of the *A. muciniphila* colonization. Distinct phylogroup-specific phenotypes of the *A. muciniphila* modulate oxygen tolerance, iron and sulfur metabolism, and bacterial aggregation differently, therefore, the genetic variations of *A. muciniphila’s* strains may influence the effect of bacterial colonization after antibiotic treatment (50). Recently, it was suggested that *A. muciniphila* phylogroups, which bear mutations in *mul* gene-cluster, lack immunomodulatory effects, but are able to colonize gut in germ-free conditions (4). It can be proposed that under antibiotic treatment, “weak” variants of the microorganism can take root and mask the immunomodulatory effects (4). Assumption about the detrimental effect of the microbiota composition disruption after antibiotics is further supported by the observed decrease in mucin expression (45, 46) upon administration of *A. muciniphila* following antibiotics. The thinning of the mucin layer allows other microorganisms to penetrate the tissue more actively and aggravate cancer-promoting intestinal inflammation.

**Figure 1:**
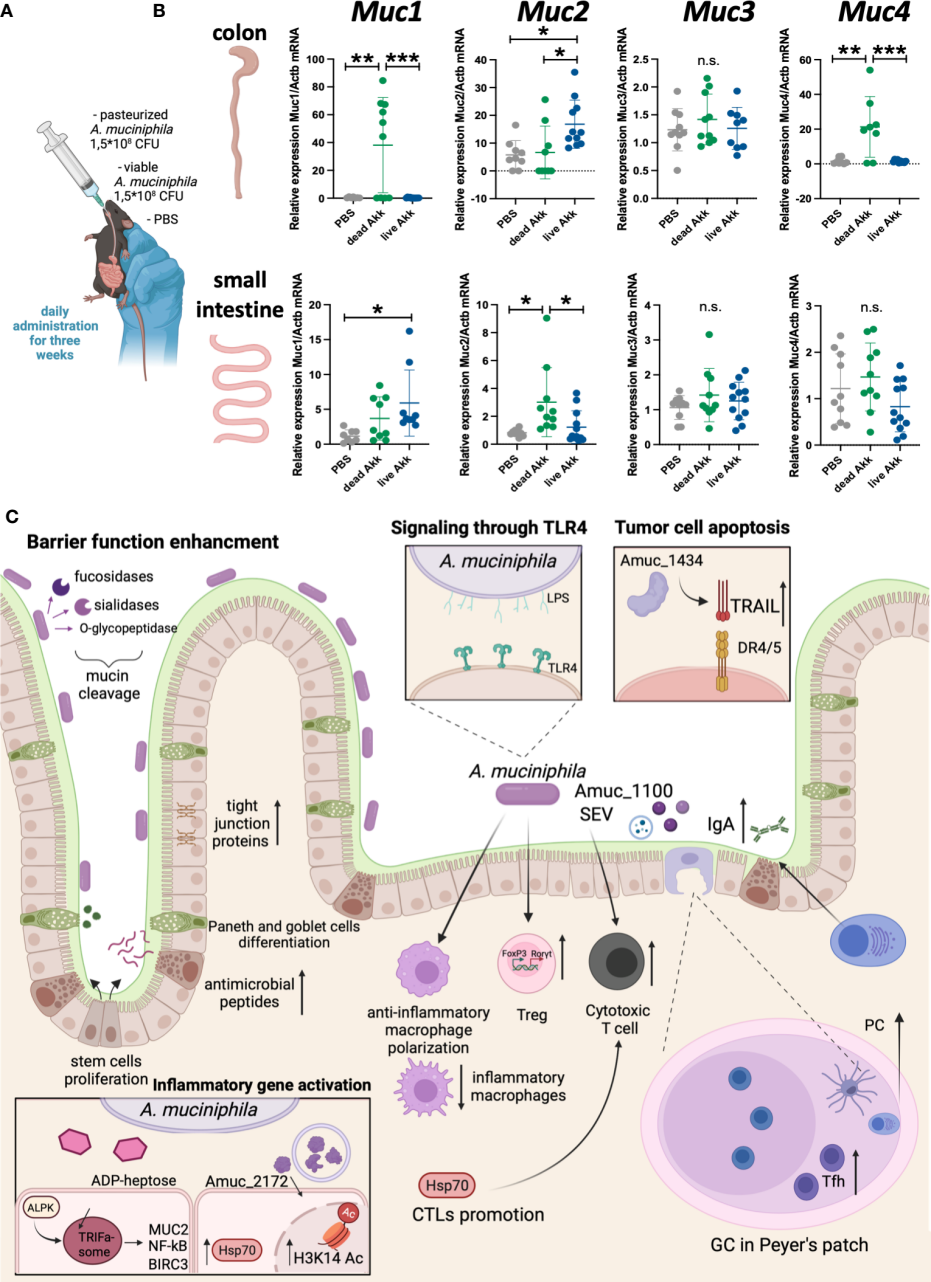
Live and pasteurized *A. muciniphila* differentially upregulate mucins expression in the gut. C57Bl/6 mice were housed in SPF conditions at the Animal Facility of the Center for Precision Editing and Genetic Technologies for Biomedicine, EIMB RAS (under the contract #075-15-2019-1660 from the Ministry of Science and Higher Education of the Russian Federation). At the age of 5-6 weeks animals of both sexes were randomly distributed between the groups and used in the experiments described below. All manipulations with animals were carried out in accordance with the protocol approved by the Bioethics Committee of the EIMB RAS (Protocol No. 3 from 27/10/22). *A. muciniphila* was grown anaerobically in the medium supplemented with porcine mucin (Sigma) and hemin (Sigma). The bacterial solution was collected at the concentration 7-8×10^7^ CFU/mL, aliquoted by 1 mL and frozen at -80°C. **(A)** Scheme of experiment. To analyze the effect of bacteria inoculation on the gene expression at steady state C57Bl/6 WT mice were randomized into three groups of 7-9 individuals and then subjected to daily *per os* administration with PBS, 1.5×10^8^ CFU of pasteurized (70°C, 30 min) *A. muciniphila* or 1.5×10^8^ CFU of live *A. muciniphila* during 3 weeks. Fresh frozen in liquid nitrogen small intestine and colon were mechanically homogenized and lysed in ExtractRNA reagent (Evrogen, Russia). RNA was isolated by guanidinium thiocyanate-phenol-chloroform method following the manufacturer’s protocol. RNA was reverse-transcribed into cDNA using RevertAid First Strand cDNA Synthesis Kit (Thermo, USA) followed by quantitative real-time PCR. qPCRmix-HS SYBR+LowROX (5X) (Evrogen, Russia). Gene expression analysis was performed using Quant Studio 6 (Applied Biosystems. USA) and the following primer set: *Actb* (F: GCGCTCTTTCAGCCTTCTTT; R: TGGCATAGAGGTCCTTGCG), *Muc1* (F: TCGTCTATTTCCTTGCCCTG; R: ATTACCTGCCGAAACCTCCT)*, Muc2* (F: CCCAGAAGGGACTGTGTATG; R: TTGTGTTCGCTCTTGGTCAG)*, Muc3* (F: TGGTCAACTGCGAGAATGGA; R: TACGCTCTCCACCAGTTCCT)*, Muc4* (F: GTCTCCCATCACGGTTCAGT; R: TGTCATTCCACACTCCCAGA). Reactions were run using the following program on the Applied Biosystems 7500: 95°C for 10 min, 40 cycles of 95°C for 15 sec, 60°C for 30 sec and 72°C for 30 sec. **(B)** Relative expression level of *Muc1*, *Muc2*, *Muc3* and *Muc4* in colon and small intestine was normalized using *Actb* and calculated as 2^-ddCt^ fold change in experimental to control group (51). Each point in a diagram represents a single mouse; mean ± SD. *P < 0,05; **P < 0,01; ***P < 0,001; ns - not significant. One-way ANOVA test was used. **(C)** *A. muciniphila* in the gut inflammation and homeostasis.

*A. muciniphila* has a direct effect on mucus production in the intestine. In this context, it appears important to establish whether different forms of bacteria - viable bacteria or pasteurized bacteria, providing distinct sets of antigens, affect the production of major mucins in the intestine. It turned out that both forms of *A. muciniphila* differently increased the expression of mucins in the gut (**Figure 1**). For example, in the colon, only pasteurized bacteria caused a significant upregulation in the expression of *Muc1* and *Muc4*, while administration of viable *A. muciniphila* alone increased the expression of *Muc2*. In the small intestine, viable, but not pasteurized, bacteria caused a slight increase in *Muc1* expression, and only pasteurized *A. muciniphila* affected the expression of *Muc2*. The expression level of *Muc3* was not affected by either form of the bacterium. Thus, the thickness of the mucus layer following *A. muciniphila* administration was significantly influenced by the form in which bacteria were administered, as well as by the tissue specificity. In the context of colorectal cancer, the thickness of the mucus layer in the large intestine is important, and the significant upregulation of mucin expression observed with the introduction of viable and pasteurized *A. muciniphila* may provide protection to tissues from microbiota invasion and inflammation (52).

In most studies administration of *A. muciniphila* protected mice from colorectal cancer. Interestingly, these studies employed experimental protocols with lower dose of *A. muciniphila* (10^7^-10^8^ CFU) (37, 43, 45, 48) and without the course of antibiotics. Some studies utilized recombinant bacterial proteins (25, 29) or secreted extracellular vesicles from *A. muciniphila* (29, 37) while Wang L. et al. used pasteurized bacterium (25).Only one study, which used a high dose of *A. muciniphila* after antibiotics, reported a protective effect of the bacterium (43). Thus, we propose that administration of the lower dose of *A. muciniphila* either in viable or pasteurized forms, as well as bacterial proteins or peptides, while maintaining the native composition of gut microbiota, has a clear protective effect on intestinal cancer, regardless of the carcinogenesis model.

## Moderation of *A. muciniphila* is the key to inflammation control

Although the mechanisms by which *A. muciniphila* controls intestinal inflammation and colorectal cancer are not fully understood, much is known about the immunomodulatory effects of the bacterium (**Figure 1С**). *A. muciniphila* is known for its mucin-reducing activity, which determines its effect on the structural components of the intestine - epithelial cells, as well as Paneth cells and goblet cells. This bacterium can enhance intestinal barrier function: *A. muciniphila* increases the expression of tight junction proteins in response to disruption of epithelial integrity *in vivo* (23, 39) and *in vitro* (24, 53). In addition, *A. muciniphila* increases the proliferation of intestinal stem cells, as well as the differentiation of Paneth and goblet cells (54) with increased antimicrobial peptides (54) and mucus production (55). In addition to accelerating the renewal of the mucus in the intestine, *A. muciniphila* activates the differentiation of Tregs in the large intestine (56) and mesenteric lymph nodes (41). Not unexpectedly, induction of protective RORγt^+^ Tregs by *A. muciniphila* is dependent on TLR4 (33, 57). *A. muciniphila*, its secretory vesicles and antigens activate a cytotoxic response in the intestine (25, 29), and, at the same time, suppress the proliferation of inflammatory macrophages (25), activate the polarization of anti-inflammatory macrophages (58). It was shown that the Amuc_1434 protein can modulate the death of tumor cells through activation of tumor-necrosis-factor-related apoptosis-inducing ligand (TRAIL) (59). *A. muciniphila* upregulates genes involved in the maintanance of intestinal barrier function via ADP-heptose-dependent activation of the ALPK1/TIFA pathway (60). Finally, *A. muciniphila* may regulate IgA production by plasma cells by affecting the number of Tfh in Peyer’s patches (61), and thus influencing the microbiota composition.

Dysbiosis is a hallmark of inflammation and intestinal cancers. *A. muciniphila* is an important component of the normal microbiota, and changes in its abundance affect the course of the disease. *A. muciniphila* is capable of inducing both proinflammatory and anti-inflammatory mechanisms. Studies on the role of *A. muciniphila* in intestinal inflammation show its protective properties in barrier restoration and control of inflammation, while data obtained in colorectal cancer models remain contradictory. Some studies indicate a decrease in tumor burden, while others report an increase in tumor growth when the bacterium is introduced. We attempted to directly compare different experimental protocols using *A. muciniphila* in various models of intestinal cancer and concluded that the introduction of large amounts of *A. muciniphila*, especially after a course of antibiotics, provokes dysbiosis, disrupts the intestinal barrier functions (62), and aggravates the inflammation that provokes cancer (46). At the same time, lower doses of the bacterium or its derivatives without prior depletion of the microbiota have a positive effect on the course of the disease. This assumption is supported by the clinical study on the correlation between the presence of *A. muciniphila* and the effectiveness of checkpoint therapy. The results of this study demonstrated that moderate, but not high *A. muciniphila* load in the stool correlated with a good prognosis (21). Thus, delicate modulation of the microbiota by *A. muciniphila* may become a promising strategy for adjunctive therapy of inflammatory bowel diseases and colorectal cancer.

## Data availability statement

The original contributions presented in the study are included in the article/supplementary files. Further inquiries can be directed to the corresponding authors.

## Ethics statement

The animal study was approved by the Bioethics Committee of EIMB RAS Protocol No. 3 from 21.09.2023. The study was conducted in accordance with the local legislation and institutional requirements.

## Author contributions

EG: Conceptualization, Data curation, Investigation, Visualization, Writing – original draft, Funding acquisition, Project administration. EG: Investigation, Writing – original draft, Methodology. MB: Resources, Writing – review & editing. OP: Resources, Validation, Writing – review & editing. AS: Methodology, Writing – original draft. AY: Methodology, Writing – original draft. EB: Conceptualization, Writing – review & editing. SN: Conceptualization, Funding acquisition, Resources, Supervision, Validation, Writing – review & editing. AK: Conceptualization, Resources, Supervision, Validation, Writing – review & editing. MD: Conceptualization, Funding acquisition, Investigation, Project administration, Resources, Supervision, Writing – review & editing.
